# A Low-Noise CMOS THz Imager Based on Source Modulation and an In-Pixel High-Q Passive Switched-Capacitor N-Path Filter

**DOI:** 10.3390/s16030325

**Published:** 2016-03-03

**Authors:** Assim Boukhayma, Antoine Dupret, Jean-Pierre Rostaing, Christian Enz

**Affiliations:** 1 Univ. Grenoble Alpes, CEA, LETI, MINATEC Campus, Grenoble F-38054, France; antoine.dupret@cea.fr (A.D.); jean-pierre.rostaing@cea.fr (J.-P.R.); 2 ICLAB, EPFL, Rue de la Maladière 71, Neuchâtel 2000, Switzerland; christian.enz@epfl.ch

**Keywords:** CMOS, terahertz direct detection, terahertz imaging, sub-millimeter wave detectors, sub-millimeter wave imaging, N-path, bandpass filter, high-Q, inductorless filter, G_m_-C, tunable, low noise

## Abstract

This paper presents the first low noise complementary metal oxide semiconductor (CMOS) terahertz (THz) imager based on source modulation and in-pixel high-Q filtering. The 31×31 focal plane array has been fully integrated in a 0.13μm standard CMOS process. The sensitivity has been improved significantly by modulating the active THz source that lights the scene and performing on-chip high-Q filtering. Each pixel encompass a broadband bow tie antenna coupled to an N-type metal-oxide-semiconductor (NMOS) detector that shifts the THz radiation, a low noise adjustable gain amplifier and a high-Q filter centered at the modulation frequency. The filter is based on a passive switched-capacitor (SC) N-path filter combined with a continuous-time broad-band G${}_{m}$-C filter. A simplified analysis that helps in designing and tuning the passive SC N-path filter is provided. The characterization of the readout chain shows that a Q factor of 100 has been achieved for the filter with a good matching between the analytical calculation and the measurement results. An input-referred noise of 0.2μV RMS has been measured. Characterization of the chip with different THz wavelengths confirms the broadband feature of the antenna and shows that this THz imager reaches a total noise equivalent power of 0.6 nW at 270 GHz and 0.8 nW at 600 GHz.

## 1. Introduction

The demand for terahertz (THz) detectors with the capability of delivering real-time imaging increases for an ever broader range of applications, such as security [1], non-destructive testing [2], medical imaging [3], pharmaceutical applications [4], soil inspection [5] and food inspection [6]. THz electronic detectors are commonly classified into coherent and incoherent detectors. Coherent detectors, also referred to as indirect or heterodyne, are sensitive to the phase and amplitude of the THz radiation. These detectors use a nonlinear device as a mixer to down-convert the THz radiation signal to a lower frequency by means of a local oscillator and sub-harmonic circuit operation [7]. Incoherent detectors, also referred to as direct detectors, are only sensitive to the intensity of the THz radiation, but not the phase and, hence, do not require any local oscillator. Incoherent detectors give less information about the THz radiation, but are better suited for building focal plane arrays. Among the THz incoherent detectors, such as bolometers [8] or Schottky barrier diodes [9], detectors based on field effect transistors (FET) emerge as a key choice for cost-efficiency, low power and on-chip integration.

THz imaging using CMOS FET-based detectors has been mainly performed using a single detector and mechanical scanning of the object [10,11]. External lock-in amplifiers have been used with modulated THz sources in order to enhance the sensitivity. The first THz CMOS camera featuring a focal plane array of 1 kpixels and operating at video frame rates has been presented in [12]. The purpose of this work is to present the first THz camera, including narrow band filtering, to enhance the sensitivity without having to use external lock-in amplifiers.

This paper explores a way to significantly increase the sensitivity of CMOS FET-based THz focal plane arrays by reducing the noise generated by the FET detector and the readout chain. The possibility of easily modulating the THz source above the flicker noise corner frequency of the FET detector and the large pixel pitch, compliant with the THz radiation wavelengths, are exploited by in-pixel integration of highly selective filtering. The bandpass filter required for this application must achieve a high-Q factor for maximum noise reduction, a tunable central frequency and an easy integration with a low layout footprint. Among the CMOS integrated filters, switched-capacitor (SC) N-path filters seem to be the best candidate to meet these conditions [13].

A 31×31 pixel CMOS THz imager operating up to 100 fps is presented. In addition to the antenna and the FET detector, each pixel of the focal plane array integrates an adjustable gain amplification stage in order to adapt the imager to the different THz sources and a tunable high-Q filter made of a combination between a passive SC N-path filter and a broadband continuous-time (CT) G${}_{m}$-C filter. The proposed N-path filter is exclusively designed with passive SC network and reaches a Q factor of 100 set by the capacitors’ ratio. This paper presents a detailed analysis of the readout circuit and particularly the narrow band filter. The analytical results are compared to simulation and measurement results complementing those already presented in [14].

This paper is organized as follows: Section 2 reviews the theory of THz detection with MOSFETs. Section 3 presents the noise reduction mechanism implemented in this work. In Section 4, the overall architecture is introduced, and the design of the building blocks is described. Section 5 presents an analytical analysis of the proposed filter; it shows that the Q factor is simply given by the capacitors’ ratio and describes how the N-path filter is optimized with a broadband G${}_{m}$-C filter. Section 6 presents the layout implementation of the proposed circuit. In Section 7, baseband measurement results to characterize the readout chain are presented together with the THz test and measurements demonstrating the operation of the proposed imaging technique.

## 2. Operation of CMOS THz Imagers

The mechanism of THz detection using MOSFETs is described by two basic theories: the plasma wave theory and the distributed resistive self mixing. In the early 1990s, Dyakonov and Shur described the channel in an idealistic ballistic FET as an electron gas that exhibits a hydrodynamic behavior similar to shallow water [15,16]. The partial differential equation (PDE) derived form the combination of Euler equation of hydrodynamic movement, the continuity equation and the dependence of the carrier sheet density on the local gate voltage led them to the description of the propagation of the plasma wave, excited by a THz radiation, in the channel. Classic resistive self-mixing is well known in RF applications for excitations below the cutoff frequency of the transistor described by a quasi-static (QS) model. This occurs when both the drain and the gate are coupled to the same radiation. The square-low dependence achieved by this self-mixing results in a DC component proportional to the square of the excitation. Under THz excitation, the non-quasi-static behavior has to be considered. Distributed resistive self-mixing extends the classic resistive self-mixing theory [17,18]. It consists of the division of the channel into small resistor-capacitor (RC) segments. Each segment is considered as a QS mixer. Plasma wave theory (in the case of non-resonant states) and distributed resistive self-mixing conduct to equivalent PDEs [19].

**Figure 1:**
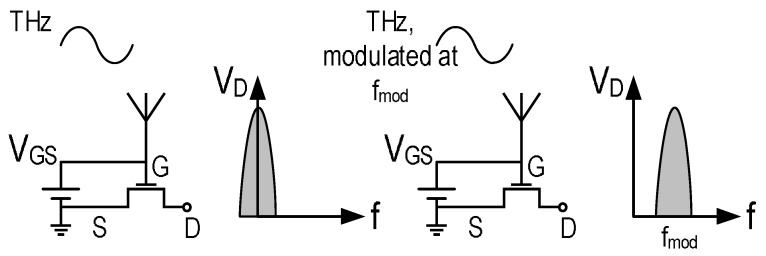
THz rectification with MOSFETs.

A practical conclusion of the theoretical approaches and test results is that a FET biased by gate-to-source voltage and excited by a THz signal between its source and gate is expected to exhibit a DC drain to source voltage that depends on the frequency and the amplitude of the THz radiation. When the THz radiation is modulated below the transition frequency of the transistor (about 80 GHz for the 130 nm process used in this work), the DC signal is shifted to the modulation frequency as depicted by Figure 1.

## 3. Noise Reduction Mechanism

**Figure 2:**
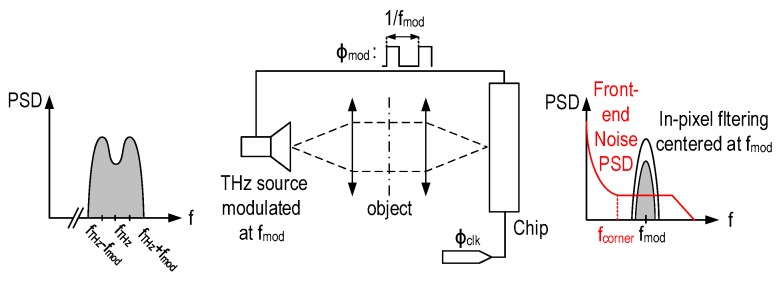
Noise reduction in CMOS THz imagers using source modulation and on-chip filtering.

The 1/f and thermal noise originating from the front-end MOSFET detector limit the signal-to-noise ratio (SNR). The 1/f noise can be avoided by shifting the signal above the flicker noise corner frequency, and thermal noise can be reduced by limiting the bandwidth. The case of THz imaging offers the possibility of controlling the source that lights the scene. Thus, in order to minimize the 1/f noise, the THz source is modulated at a frequency $f_{mod}$ higher than the front-end flicker noise corner frequency. A filter centered at the modulation frequency is then applied to the signal at the output of the MOSFET detector rejecting the 1/f noise, as depicted by Figure 2. This mechanism acts as chopper stabilization [20]. Based on simulation results of the front-end MOSFET, the flicker noise corner frequency is about 10 kHz. Hence, a modulation at 100 kHz is enough to cancel the 1/f noise. The thermal noise variance is proportional to the in-pixel filtering bandwidth. Hence, narrow band filtering is highly required. However, the filtering center frequency must match with the modulation frequency. In order meet this condition, the modulation phase $\Phi_{mod}$ is generated on chip in order to synchronize the filter with the modulation.

The modulation of the THz source can be performed mechanically using choppers or electrically using on-off modulation. It has been shown experimentally that the modulation increases the sensitivity of MOSFET-based THz detectors, even without implementing any bandpass filter after the front-end MOSFET [11,12]. Moreover, high integration times of a few milliseconds can be reached while achieving high frame rates, even with focal plane arrays thousands of times larger than the state-of-the-art. Thus, very high selective filtering can be implemented without being limited by the filter’s rise time. The in-pixel readout chain design based on this noise reduction mechanism is detailed in the following section.

## 4. Architecture of the Low Noise CMOS THz Imager

### 4.1. Overall Architecture

**Figure 3:**
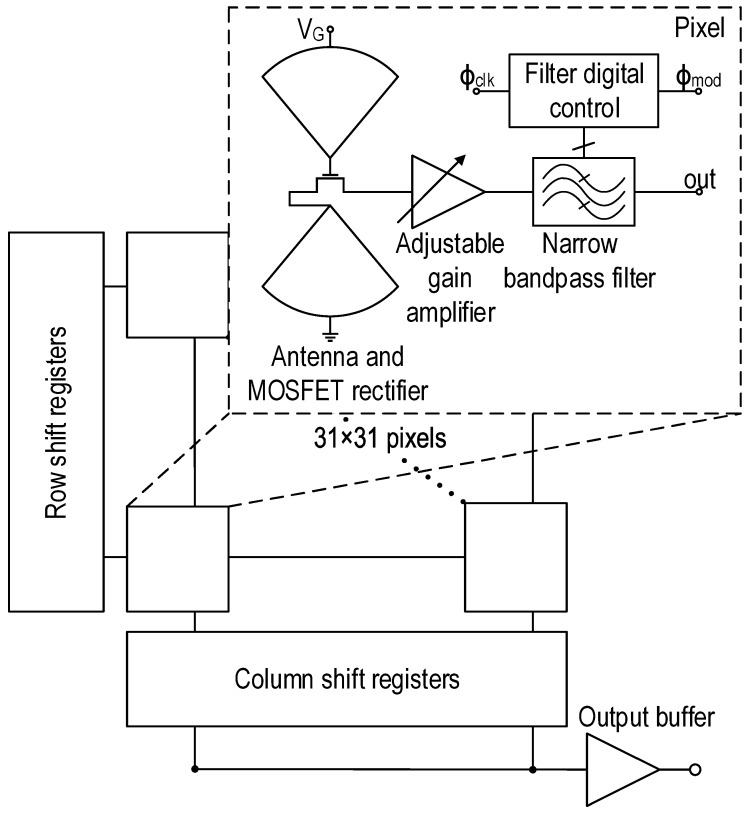
Overall block diagram of the CMOS THz imager.

The presented THz camera is an array of 31×31 pixels. The overall block diagram of the imager, as well as the pixels are shown in Figure 3. Row and column shift registers are used to address simultaneously the pixels of the array. Each pixel comprises an antenna coupled to a MOSFET detector, a low noise adjustable-gain amplifier and a high-Q filter. As discussed in Section 3, the pixel includes a digital block that controls the filter and generates the modulation signal $\Phi_{mod}$. In this way, the modulation frequency of the THz source is locked with the center frequency of the filter.

### 4.2. In-Pixel Antenna and Rectifier

**Figure 4:**
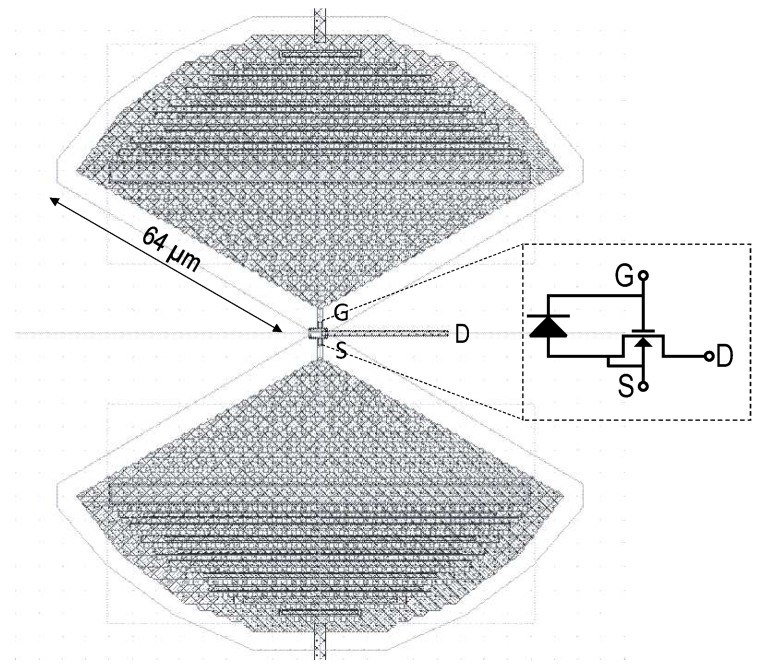
Layout view of the in-pixel THz antenna and MOSFET detector.

Bow tie antennas have been chosen in this design for their broadband characteristics. In addition, these antennas are relatively easy to integrate in the back-end metal of the standard CMOS process compared, for instance, to slot antennas. Log periodic and spiral periodic antennas, known for their ultra-broadband characteristics, are not easy to integrate under the restrictions and layout design rules. Figure 4 shows a layout view of the in-pixel antenna coupled to the NMOS used as a detector. The dimensions of the bow tie antenna have been chosen based on the measurements performed previously in [10,21] in order to optimize the gain and the impedance matching with the NMOS detector. The radius of the antenna is 64μm with an angle of $\frac{2}{3}\pi$. The two terminals of the antenna are connected respectively to the gate and source of an NMOS transistor used as a FET detector. The two terminals of the antenna are also used to bias the NMOS by applying a gate-to-source voltage. A protection P-N junction diode is connected to the antenna in parallel to the NMOS.

### 4.3. In-Pixel Amplification

**Figure 5:**
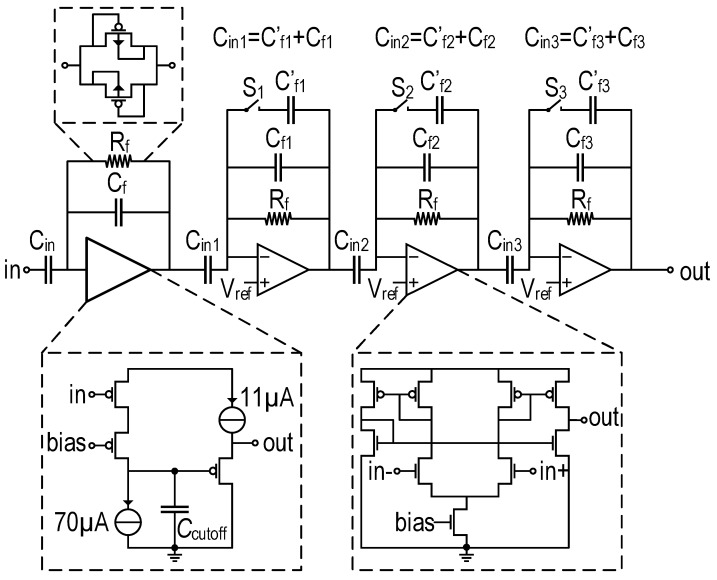
Schematic of the in-pixel adjustable gain amplifier.

The baseband voltage generated at the drain node of the MOSFET detector is weak. In fact, the responsivity is of the order of a few hundreds of V/W. Standard electrical THz sources deliver Gaussian THz beams of a few mW in the focal plane; thus, at each pixel, the signal at the output of the detector is, at the best, expected to be of the order of a few hundreds of μV. Such a weak signal requires a low noise amplifier at the output of the detector. In addition, this amplifier reduces the load of the detector to allow faster modulation. The amplification stage comprises a low noise closed-loop amplifier cascaded with three variable closed loop gain stages, as shown by Figure 5.

The first stage is designed to provide a closed loop gain of 32 dB. In order to achieve a low noise performance, this amplifier relies on a minimum number of transistors. It consists of a single-ended amplifier made of a cascode common source stage to provide the gain and a source follower stage for a low output impedance. Since high speed is not required for this application, PMOS transistors have been used for their lower 1/f noise. The input MOSFET of the amplifier has been designed with a large W/L of 1600 in order to achieve a high open-loop gain with minimum noise. The current sources have been designed with compact MOS devices delivering 70μA to the input cascode stage and 11μA to the source follower stage. The capacitor $C_{cutoff}$ of 4 pF limits the bandwidth at the output of the common source stage. It is realized with a MOS capacitance. A high feedback resistor of several GΩ has been used to supply the gate bias of the common source stage. As shown in Figure 5, it has been realized with two back-to-back P-type metal-oxide-semiconductor (PMOS) diodes.

The next amplifying stages consist of three operational transconductance amplifiers (OTAs) delivering, consecutively, a closed-loop gain of 2, 5 and 10. The gain of each stage can be set to unity using a switched capacitor, as shown in Figure 5. The cascaded amplifiers provide a gain that can be varied over a large range in order to adapt the imager to different THz sources. Each of the three stages of the variable gain amplifier features a simulated phase margin of $60^{\circ }$, a gain margin of 40 dB, a gain bandwidth of 10 MHz and a current consumption of 15μA.

Based on AC noise simulations of the amplification stage, the input-referred noise PSD of the detector and amplifier is about 5 nV/$\sqrt{Hz}$ at 100 kHz and the 1/f noise corner frequency is about 10 kHz.

### 4.4. In-Pixel Filtering

The aim of this stage is to select the spectral component centered at the modulation frequency of the THz source. A high-Q filter is needed to achieve high noise rejection, as discussed previously. In addition, the filter has to be designed to feature a small layout footprint and a low mismatch in order to achieve low pixel-to-pixel non-uniformity. It is difficult to match those conditions using conventional analog techniques. Active SC circuits offer a good alternative; however, they rely on high gain operational amplifiers. In addition to significant power consumption, such active circuits can suffer from stability and sensitivity problems. Consequently, the high-Q filtering is implemented using an N-path filter. N-path filters allow the design of bandpass and band-reject filters without the use of inductors. The bandwidth of the N-path filter is independent of the center frequency, which is only determined by a clock frequency, which makes it easily tunable and robust against mismatch and process variations. The N-path filter also offers a fundamental advantage: The THz modulation frequency and the central frequency of the filter can be generated by the same digital block, which makes them synchronized. This condition is not easy to meet with conventional CT filters.

**Figure 6:**
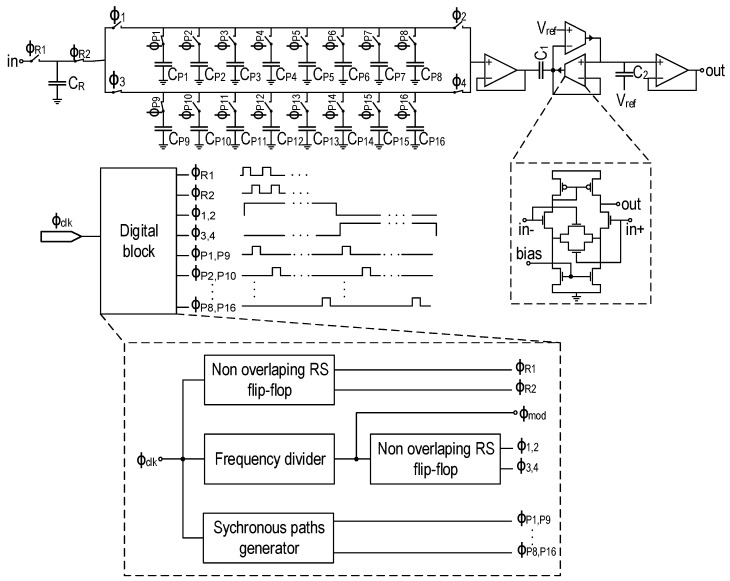
Schematic of the in-pixel high-Q filter.

In this design, a passive N-path filter, exclusively designed with switched capacitors, has been combined with a CT G${}_{m}$-C filter. Figure 6 shows the schematic of the N-path and G${}_{m}$-C filters. The SC network of the N-path filter is made of an input capacitor $C_{R}$ of 40 fF and a network of 16 identical capacitors $C_{P1}...C_{P16}$ of 1.3 pF each. The input capacitor $C_{R}$ replaces the input resistance in a classical passive SC N-path filter [22]. It samples the signal with a frequency of $16f_{mod}$. The 16 capacitors $C_{P}$ are cycled with the central frequency of the filter. A digital block composed of frequency dividers generates the different non-overlapping phases controlling the switches out of an external clock signal running at $16f_{mod}$. The corner frequency of the noise at the output of the amplification stage is about 10 kHz; hence, modulation frequencies around few a hundreds of kHz are enough to filter out the 1/f noise. Consequently, the N-path filter is operated with low frequencies, which makes the design of the digital control block and the SC network free of the constraints related to the high frequency operation, like the switches non-ideality and the phase noise. As will be discussed in the next section, a CT G${}_{m}$-C filter is cascaded with the N-path filter for noise optimization. The transconductors are implemented using degenerated operational transconductance amplifiers (OTAs) for good linearity [23].

## 5. Analysis of the High-Q Filter

### 5.1. Passive SC N-Path Filter

**Figure 7:**
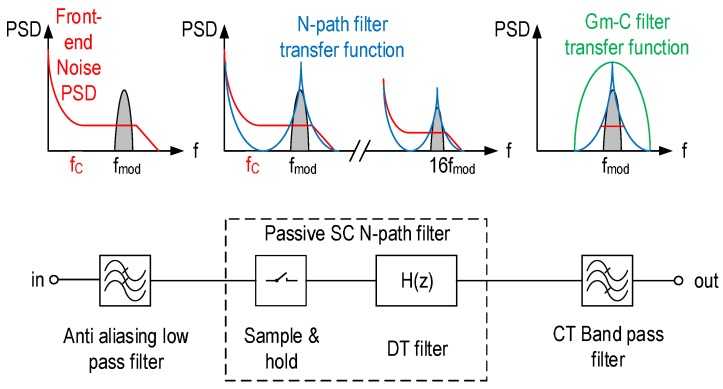
Signal processing block diagram of the readout chain with a spectrum analysis of the filter.

Exhaustive analysis of N-path filters based on switched passive RC networks have been presented in [13,22,24,25]. Here, we aim to derive the transfer function of the passive SC network in an intuitive way and give a simple expression of the transfer function. N-path filters are generally analyzed as linear periodically time-variant systems. The N-path filter design presented in this paper is a discrete-time (DT) system used to process a CT signal. Figure 7 presents the in-pixel readout chain as a signal processing block diagram. The input signal is band-limited by the amplification stage that acts as an anti-aliasing filter. The CT signal is converted to a DT signal at the input of the N-path filter, processed in DT and then reconstructed at the output using the bandpass G${}_{m}$-C filter.

**Figure 8:**
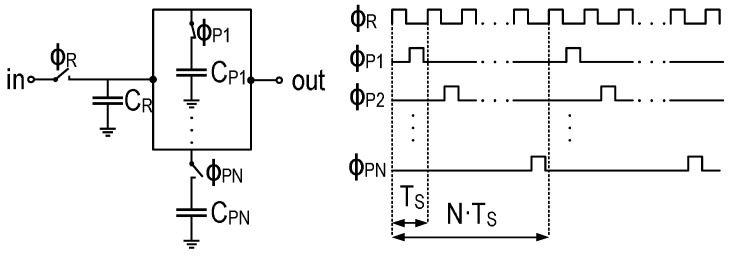
Simplified schematic of the passive switched-capacitor (SC) N-path filter with its timing diagram.

The DT N-path filter is analyzed using the Z transform. Figure 8 shows a simplified schematic of the passive SC N-path filter. We assume that the on resistance of the switches is low enough for a complete settling of the signal during the sampling period $T_{s}$. The filter is made of an input capacitor $C_{R}$ and *N* identical capacitors $C_{P}$. The input CT signal is sampled and held in $C_{R}$ with a sampling period $T_{S}$, which is set to be equal to $\frac{1}{N\cdot f_{mod}}$ in order to match the filter’s central frequency with the modulation frequency $f_{mod}$ of the THz source. The capacitors $C_{P1}...C_{PN}$, of the N paths, are cycled with a period of $N\cdot T_{s}$. At the end of the sampling period $\left(n-1\right)T_{s}$, the input capacitor $C_{R}$ holds the voltage $V_{in}\left(\left(n-1\right)T_{s}\right)$. During the next switching period, one of the capacitors $C_{P1}...C_{PN}$ is connected to the output. It holds the voltage $V_{out}\left(\left(n-N\right)T_{s}\right)$ from the previous cycling period ($\left(n-N\right)T_{s}$). Both capacitors are connected to the output node. Thus, they share their charges, resulting in an output voltage given by:

(1) $$V_{out}\left(nT_{s}\right)=\frac{C_{R}V_{in}\left(\left(n-1\right)T_{s}\right)+C_{P}V_{out}\left(\left(n-N\right)T_{s}\right)}{C_{R}+C_{P}}$$

where $C_{P}=C_{P1}=...=C_{PN}$. Consequently, the linear constant-coefficient difference (LCCD) equation of the N-path filter is given by:

(2) $$V_{out}\left(n\right)=\frac{C_{R}}{C_{R}+C_{P}}V_{in}\left(n-1\right)+\frac{C_{P}}{C_{R}+C_{P}}V_{out}\left(n-N\right)$$

This equation leads to the expression of the Z transform of the transfer function of the filter:

(3) $$H_{N}\left(z\right)=\frac{V_{out}\left(z\right)}{V_{in}\left(z\right)}=\frac{\frac{C_{R}}{C_{R}+C_{P}}z^{-1}}{1-\frac{C_{P}}{C_{R}+C_{P}}z^{-N}}$$

The equivalent transfer function of the DT N-path filter in the Fourier domain is obtained by substituting $e^{-j2\pi \frac{f}{Nf_{mod}}}$ for *z*, since the unity circle is included in the region of convergence of H(z) given by $|z|>{\left(\frac{C_{P}}{C_{R}+C_{P}}\right)}^{\frac{1}{N}}$.

(4) $$H_{N}\left(f\right)=\frac{V_{out}\left(f\right)}{V_{in}\left(f\right)}=\frac{\frac{C_{R}}{C_{R}+C_{P}}e^{-j2\pi \frac{f}{Nf_{mod}}}}{1-\frac{C_{P}}{C_{R}+C_{P}}e^{-j2\pi \frac{f}{f_{mod}}}}$$

Thus,

(5) $$|H_{N}{\left(f\right)|}^{2}=\frac{{\left(\frac{C_{R}}{C_{R}+C_{P}}\right)}^{2}}{1+{\left(\frac{C_{P}}{C_{R}+C_{P}}\right)}^{2}-2\left(\frac{C_{P}}{C_{R}+C_{P}}\right)cos\left(2\pi \frac{f}{f_{mod}}\right)}$$

Equation (5) suggests that the frequency response of the passive SC filter presented in Figure 8 is a periodic bandpass filter centered at frequencies $kf_{mod}$ for $k<\frac{N}{2}$. The 3-dB bandwidth $\Delta f_{3dB}$ can be expressed as ($2(f_{3dB}-f_{mod})$) with $|H\left(f_{3dB}\right){|}^{2}=\frac{1}{2}$. The Q factor is then expressed as:

(6) $$Q=\frac{f_{mod}}{\Delta f_{3dB}}$$

and the expression of $f_{3dB}$ can be derived as

(7) $$cos\left(2\pi \frac{f_{3dB}}{f_{mod}}\right)=1-\frac{1}{2}{\left(\frac{C_{R}}{C_{P}}\right)}^{2}\frac{1}{1+\frac{C_{R}}{C_{P}}}$$

In order to simplify Equation (7), we consider the case of a high-Q factor for which:

(8) $$f_{3dB}-f_{mod}=\frac{1}{2}\Delta f_{3dB}\ll f_{mod}$$

In this case:

(9) $$cos\left(2\pi \frac{f_{3dB}}{f_{mod}}\right)≃1-\frac{1}{2}{\left(\pi \frac{\Delta f_{3dB}}{f_{mod}}\right)}^{2}$$

The expression of the Q factor is obtained by combining Equations (7) and (9), resulting in:

(10) $$Q=\pi \frac{C_{P}}{C_{R}}\sqrt{1+\frac{C_{R}}{C_{P}}}$$

In the case of a high-Q factor, $\frac{C_{P}}{C_{R}}$ must be higher than one. In this case, Equation (10) simplifies to:

(11) $$Q≃\pi \frac{C_{P}}{C_{R}}$$

**Figure 9:**
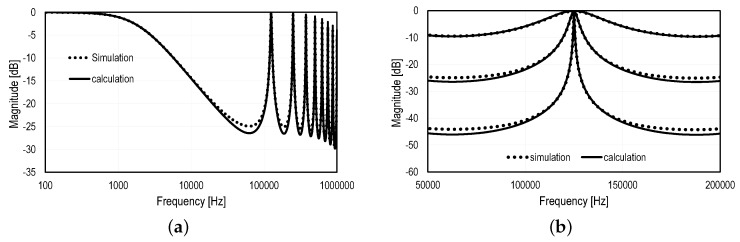
Simulated transfer function of the N-path filter from Figure 8 with Spectre RF${}^{©}$ compared to the calculated transfer function from Equation (12) for N=16 and $f_{mod}=125\;$ kHz. (**a**) Both transfer functions, in the Log frequency scale with $\frac{C_{P}}{C_{R}}=100$, and the impact of the sinc lobes; (**b**) a zoom in a linear scale for $\frac{C_{P}}{C_{R}}$ set to 1, 10 and 100.

We conclude that the passive SC N-path filter depicted in Figure 8 has a periodic frequency response corresponding to a periodic bandpass filter centered at frequencies $kf_{mod}$ for *k* lower than $\frac{N}{2}$, and the quality factor of this filter depends only on the ratio $\frac{C_{P}}{C_{R}}$. Based on the signal processing block diagram of Figure 7, the power spectral density (PSD) of the signal at the output of the N-path filter can be expressed as:

(12) $$S_{out}\left(f\right)=sinc^{2}\left(\pi N\cdot f_{mod}\right)\cdot {\left|H_{N}\left(f\right)\right|}^{2}\cdot \sum_{n=-\infty }^{+\infty }S_{in}\left(f-n\cdot N\cdot f_{mod}\right)$$

where $S_{in}$ and $S_{out}$ refer respectively to the PSD at the input and output of the SC N-path filter. In order to validate the derivation of the transfer function at the output of the N-path filter, the passive SC network shown in Figure 8 has been simulated using Spectre RF${}^{©}$. Figure 9 shows the simulated transfer function compared to the calculation resulting from Equation (12) for N=16 and $f_{mod}=125\;$ kHz. Figure 9a shows the frequency response for $\frac{C_{P}}{C_{R}}=100$ in the log frequency scale and demonstrates the good matching between the simulation results and Equation (12). Figure 9b shows the frequency response of the passive SC N-path filter for $\frac{C_{P}}{C_{R}}$ set to 1, 10 and 100. The latter figure shows the frequency response in a linear scale with a zoom into the central frequency of the filter, which validates Equation (10), suggesting that the Q factor only depends on the ratio $\frac{C_{P}}{C_{R}}$.

### 5.2. Optimization with a G${}_{m}$-C Filter

As mentioned in the previous section, the passive SC N-path filter has a periodic frequency response. The baseband spectral component and low frequency noise are therefore not rejected, as depicted in Figure 7. Thus, for an optimized filtering, an additional wide band CT bandpass filter with a lower Q factor has to be applied to the output signal in order to filter out the out-of-band spurious signals and noise. As illustrated in Figure 7, the second filter selects the spectral component centered at $f_{mod}$ and filters out the 1/f noise. A Q factor of one is enough for the second CT G${}_{m}$-C filter, which is implemented as shown in Figure 6. The square magnitude of the frequency response of this filter is given by:

(13) $$|H_{G_{m}-C}{\left(f\right)|}^{2}=\frac{1}{1+Q^{2}{\left(\frac{f}{f_{0}}-\frac{f_{0}}{f}\right)}^{2}}$$

where the quality factor Q is given by:

(14) $$Q=\sqrt{\frac{g_{m1}}{C_{1}}\cdot \frac{C_{2}}{g_{m2}}}$$

In this design, $g_{m1}=g_{m2}=g_{m}$ and $C_{1}=C_{2}=C$. Thus, Q=1. The center frequency $f_{0}$ of the G${}_{m}$-C filter is given by:

(15) $$f_{0}=\frac{1}{2\pi }\sqrt{\frac{g_{m1}\cdot g_{m2}}{C_{1}\cdot C_{2}}}=\frac{1}{2\pi }\frac{g_{m}}{C}$$

The latter is set to be equal to the modulation frequency $f_{mod}$. It is tuned using the bias voltage of the transconductors that determines the value of $g_{m}$ [23].

## 6. Circuit Implementation

**Figure 10:**
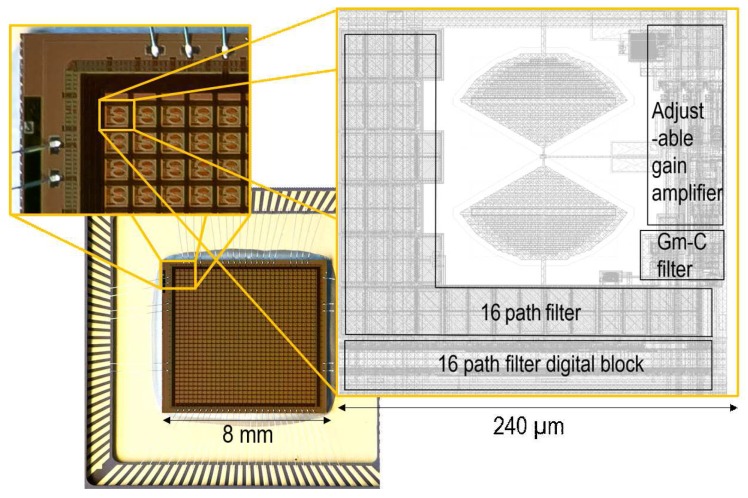
Chip micrograph with a zoom on the pixel layout.

The test chip is fabricated in a standard 130 nm P5M CMOS process. The 31×31 pixels array with the row and column circuitry occupy an area of 8 mm× 8 mm with a pixel pitch of 240μm. Both analog and digital parts of the readout chain are powered with a 1.2-V supply. Figure 10 shows the chip micrograph with a zoom onto the layout of the pixel. Note that 40% of the pixel area is dedicated to the bow tie antenna integrated using four metal layers. The rest contains the adjustable gain amplifier, the N-path and G${}_{m}$-C filters, a buffer stage, as well as the digital block generating the different phases controlling the switches of the SC N-path filter. Among the readout chain, the N-path filter has the largest layout footprint due to its 16 capacitors of 1.3 pF each integrated using MOS capacitors.

## 7. Test and Characterization

The chip test follows two steps: first, a baseband characterization of the readout circuit is performed in order to measure the voltage gain and validate the theoretical and simulation analysis of the filter. The readout chain total RMS noise voltage $V_{n,RMS}$ is then measured. The second step consists of demonstrating the operation of the whole readout mechanism with different THz sources.

### 7.1. Baseband Characterization

**Figure 11:**
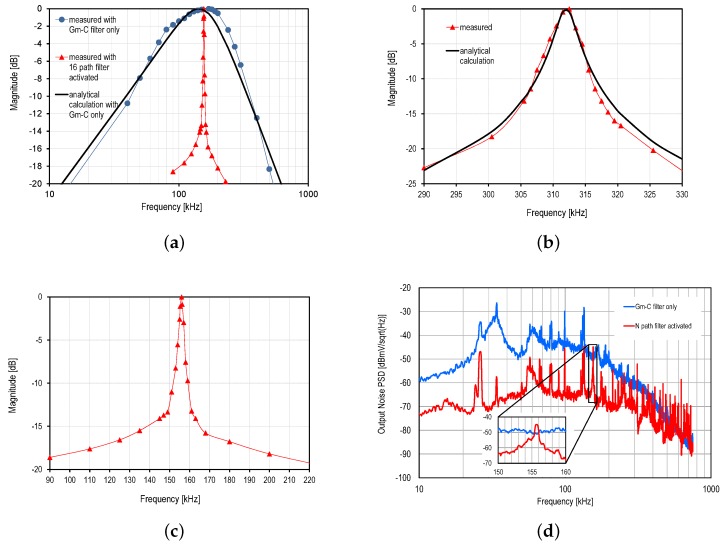
Characterization of the in-pixel baseband circuit with a measured readout chain gain of 58 dB. (**a**) The transfer function (log frequency scale) when the N-path filter is bypassed, compared to the analytical calculation and with the N-path filter activated; (**b**) the measured transfer function (linear frequency scale) when both filters are activated and centered at 312 kHz compared to the analytical calculation; (**c**) measured transfer function (linear frequency scale) when both filters are activated and centered at 156 kHz; (**d**) the output noise PSD obtained when both filters are activated and when the N-path filter is bypassed with a zoom onto the center frequency of the N-path filter.

The first pixel of the matrix contains an analog input directly after the THz antenna and FET detector. This input has been used to characterize the readout circuit. The frequency response of the readout chain is obtained by applying a frequency sweep of an input sine wave. Figure 11a shows the normalized frequency response of the filter when it is centered at 156 kHz in a log frequency scale. It shows the measured frequency responses of the filter when the N-path filter is activated and bypassed. The frequency response when the N-path filter is bypassed is compared to the calculated transfer function obtained from Equation (13) and given by:

(16) $${\left|H\left(f\right)\right|}^{2}=\frac{|H_{G_{m}-C}{\left(f\right)|}^{2}}{1+{\left(\frac{f}{f_{c}}\right)}^{2}}$$

where $f_{c}$ is the cutoff frequency of the output buffer. Figure 11b,c shows, in a linear frequency scale, the frequency response normalized to the peak gain when the N-path filter is activated and centered at 312 kHz and 156 kHz, respectively. The frequency response when the N-path filter is centered at 312 kHz is compared to the calculated transfer function obtained from Equations (12) and (13) given by:

(17) $${\left|H\left(f\right)\right|}^{2}=\frac{sinc^{2}\left(\pi N\cdot f_{mod}\right)\cdot |H_{N}\left(f\right){|}^{2}\cdot {\left|H_{G_{m}-C}\left(f\right)\right|}^{2}}{1+{\left(\frac{f}{f_{c}}\right)}^{2}}$$

for N=16 and $\frac{C_{P}}{C_{R}}=100/\pi$. Figure 11a–c demonstrate the extremely high selectivity of the filter having a Q factor of 100. It also shows the excellent match between the theoretical model presented in Section 5 and the measurement results. Note that a loss of 8% has been measured when the N-path filter is activated due to the passive implementation of the SC N-path filter.

Figure 11d shows the broadband noise power spectral density (PSD) measured with a spectrum analyzer at the output of the readout chain when the N-path filter is activated and bypassed. The broadband noise PSD is clearly shaped by the frequency response of the G${}_{m}$-C filter when the N-path filter is bypassed. For this measurement, the N-path filter has been centered at 156 kHz. Figure 11d shows that when the N-path filter is activated, the noise PSD is reduced by 20 dB out of the narrow SC N-path filter band. The output RMS noise voltage has been calculated by integrating the measured PSDs over the frequency range [0 : 500 kHz] (the bandwidth of the output buffer) resulting in 0.2 mV when the N-path filter is activated and 1.9 mV when bypassed. These measurement have been performed with a measured readout chain gain of 58 dB (including the loss of the N-path filter). The corresponding total input-referred noise is as low as 0.2μV. The simulation results of the amplification stage show that the input referred noise PSD of the detector and amplification stages is about 5 nV/$\sqrt{Hz}$ at 100 kHz, and the 1/f noise corner frequency is about 10 kHz. The simulations also showed that 74% of the input-referred readout chain noise originates from the MOSFET front-end and 20% from the amplification stage. Thus, the flicker noise is expected to be canceled, which is a big advantage of the proposed technique. Assuming a filter Q of about 100, the bandwidth is about 1.56 kHz for a center frequency of 156 kHz, resulting in a thermal noise voltage of 197 nV. The measurement results show that the input referred noise is 248 nV, which is close enough to the rough estimation that does not include the additional noise due to the off-chip buffers and measurement setup.

### 7.2. THz Characterization

A characterization of the presented THz imager has been performed with different active sources in order to validate the readout scheme depicted in Figure 2 and to measure the responsivity of the imager with a modulated THz radiation. The imager responsivity is measured as the ratio between the total output voltage of all of the pixels of the imager and the power of the THz radiation incident to the imager position. It is expressed in V/W as:

(18) $$R_{v}=\frac{\Sigma_{i=1}^{961}V_{out,i}}{P_{THz}}$$

where $V_{out,i}$ refers to the voltage amplitude at the output of the *i*-th pixel of the imager and $P_{THz}$ refers to the measured THz power incident to the chip area. Note that this measurement method gives an absolute responsivity taking into account the fill factor and the antenna directivity.

The sensitivity of the imager is then expressed as the total noise equivalent power $NEP_{tot}$ in Watts:

(19) $$NEP_{total}=\frac{V_{n,RMS}}{R_{v}}$$

The $NEP_{total}$ represents the amount of THz power corresponding to an SNR of one without having to take into account any other off-chip considerations. Note that $NEP_{total}$ differs from the NEP. The NEP, which is given in pW/$\sqrt{Hz}$, better describes the noise performance of pixels only comprising a detector and low-noise amplifier and for which the bandwidth control is performed off-chip by means of a lock-in amplifier, for instance [11]. In this work, the selective filtering is performed in-pixel. The global performance in this case is given in $NEP_{total}$, as reported in [12]. The NEP value can be obtained from the noise spectral density at the modulation frequency, which is also the center frequency of the filter.

**Figure 12:**
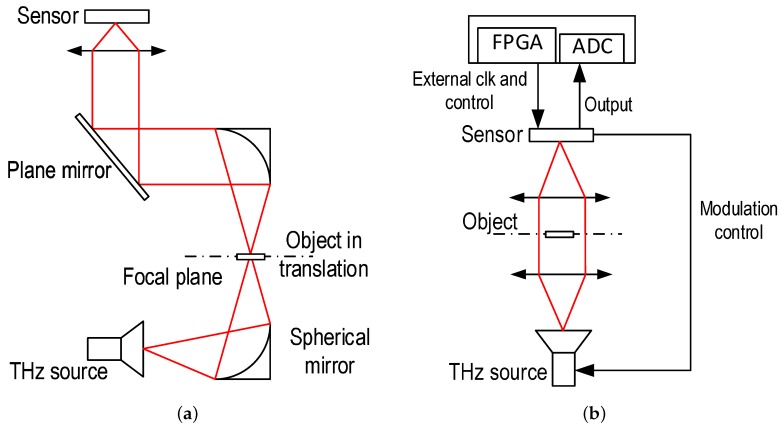
Experimental setup used with (**a**) the 2.5 THz laser gas source; and (**b**) with the multiplying chain sources in the range [200:600] GHz.

Two continuous-wave (CW) frequency multiplying chains are used to generate the THz radiation between 200 GHz and 600 GHz. The experimental setup used with these CW sources is depicted in Figure 12. The THz sources are modulated electrically (on/off modulation) using the modulation phase $\Phi_{mod}$ generated by the on-chip digital block in order to be synchronous with the center frequency of the in-pixel high selective filtering. Two lenses sharing the same optical axis are positioned between the THz source and the tested imager. A distance close to the lenses’ focal length separates the CW THz source and the imager from the corresponding lenses in order to make sure that most of the THz power generated by the CW source is projected on the tested focal plane array. A power meter based on a Schottky barrier diode is then used to measure the total available power at the chip position. The CW 200 GHz source delivers a power of 2 mW at the chip position, and the one used to generate THz radiations in the range of [270–600 GHz] delivers a power of 1 mW at 270 GHz and 0.5 mW at 600 GHz.

A gas laser 2.5 THz source has also been used in order to characterize the THz imager at the higher edge of the THz band. Unfortunately, electrical modulation is not possible with such a source, and the mechanical chopping operates at frequencies below hundreds of Hertz. Thus, the characterization method described previously could not be applied in this case.

**Figure 13:**
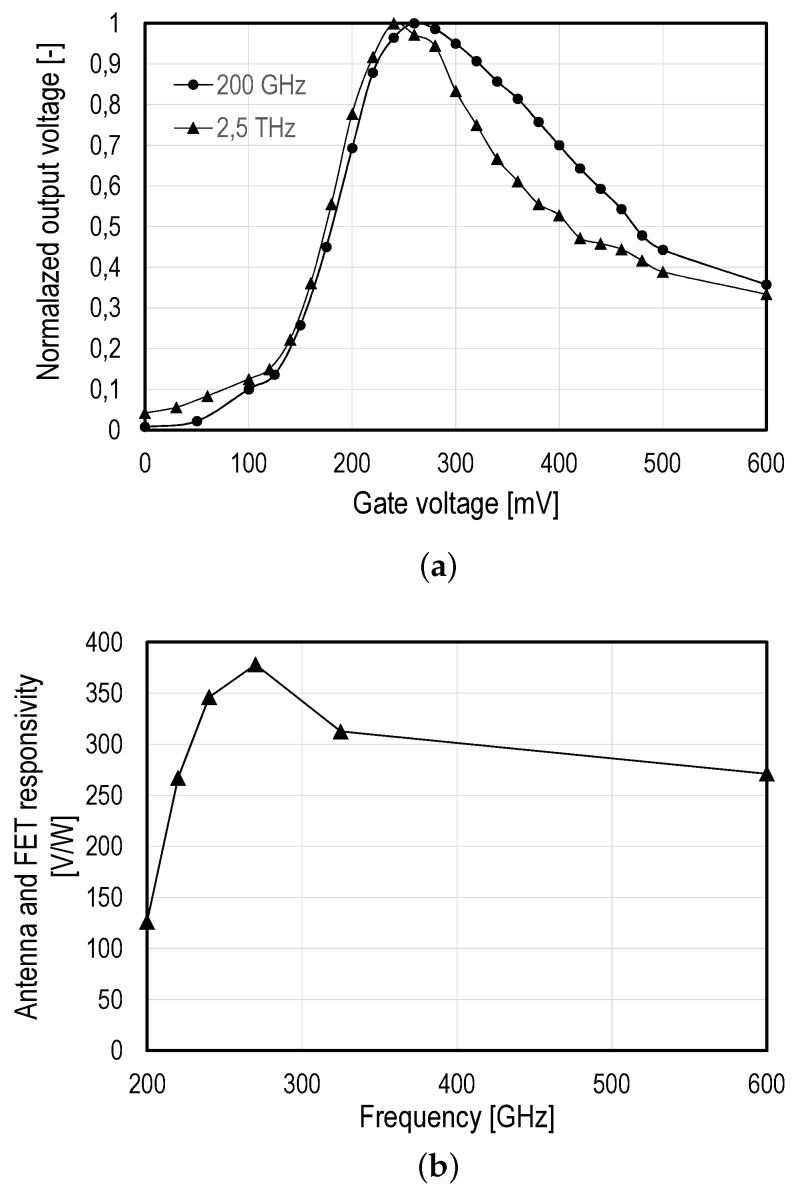
THz characterization of the sensor: (**a**) the amplitude of the output voltage, normalized to its maximum, over the gate bias for THz radiations at 200 GHz and 2.5 THz; (**b**) the measured responsivities for frequencies between 200 GHz and 600 GHz.

First, the test pixel was used in order to identify the gate bias voltage corresponding to the maximum responsivity. Figure 13a shows the voltage at the output of the MOSFET detector (between the drain and source), normalized to its maximum value, as a function of the gate bias voltage (gate-to-source). For THz radiations of 200 GHz and 2.5 THz, the amplitude of the output voltage reaches its maximum for a gate bias of 0.25 V.

Figure 13b shows the responsivities obtained using Equation (18) with the CW multiplying chain THz sources modulated at 156 kHz with the imager readout chain set to 58 dB. It was not possible to obtain measurements in the full 3-dB bandwidth of the antenna, but this measurement shows that it is larger than 400 GHz and confirms the broadband characteristic of the bow tie antenna. These measurements were used to calculate $NEP_{total}$ based on Equation (19). The $NEP_{total}$ values are 0.6 nW at 270 GHz and 0.8 nW at 600 GHz.

In order to obtain the NEP value in pW/$\sqrt{Hz}$, the measured noise PSD at 156 kHz (shown in the inset of Figure 11d) is used to obtain the output noise in V/$\sqrt{Hz}$; this value is then divided by $R_{v}$ in order to calculate the NEP. From the inset of Figure 11d, showing a zoom onto the center frequency of the filter, the measured output noise at the center frequency is -4 dBmV/$\sqrt{Hz}$, corresponding to 5.6 μV/$\sqrt{Hz}$. The resulting NEP value is then 18.7 pW/$\sqrt{Hz}$ at 270 GHz. Similarly, a NEP of 25.9 pW/$\sqrt{Hz}$ is obtained at 600 GHz accounting for the same PSD value and a slightly reduced $R_{v}$ of 216 kV/W.

**Figure 14:**
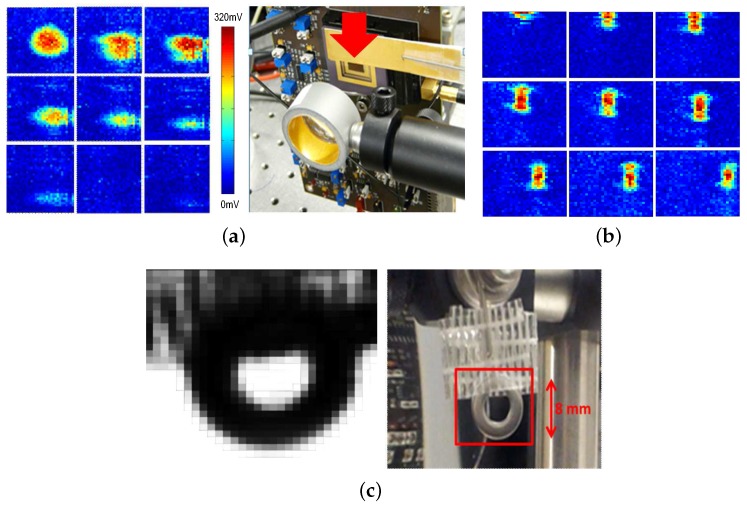
(**a**) Video sequence, obtained at 100 fps, of a copper ruler passing in front of a 200-GHz beam of 2 mW measured at the imager position and modulated at 156 kHz; (**b**) video sequence, obtained at 100 fps, when translating vertically then horizontally the imager exposed to a 270-GHz source with a rectangular waveguide; the THz source is modulated at 156 kHz and delivers 0.5 mW at the sensor position; (**c**) image of a metallic ring held with tape obtained with 2.5 THz using the setup of Figure 12, the antenna and FET detector of one pixel and a lock-in amplifier.

Figure 14a shows the image of the 200-GHz Gaussian beam. It can be noticed that the beam is not large enough to cover the whole imager. Thus, making good images without mechanical scanning is still difficult with such levels of THz radiation power. Figure 14a also shows images from a video sequence obtained, at a frame rate of 100 fps, when passing a copper ruler in front of the imager to reflect the THz beam. Figure 14b shows images from a video sequence, obtained at 100 fps, when translating vertically then horizontally the imager exposed to a 270 GHz source with a rectangular waveguide. The THz source is modulated at 156 kHz and delivers 0.5 mW. The characterization of the imager with the 2.5-THz source was not possible for the reasons mentioned above; thus, only the antenna and FET detector part of one pixel from the array was used in order to obtain an image by performing a mechanical scan. Figure 12a shows the experimental setup used for this operation. The object is positioned in the focal point between two spherical mirrors. The THz radiation is then collimated by the second mirror and focused on the sensor. Figure 14c shows the image of a metal ring held with tape, using the setup described above, by mechanically scanning the object in the focal plane between the two spherical mirrors. The THz power measured at the sensor position is 10 mW. The readout was performed with a lock-in amplifier and a mechanical chopping of 290 Hz.

The global performance of the presented THz image sensor is summarized in Table 1 and compared to recently-reported THz focal plane arrays. The power consumption of the presented sensor is higher than other works. This is mainly due to the power consumption of the closed loop amplifiers. The power consumption of the digital block controlling the SC N-path filter can be reduced by sharing the digital control signals between multiple pixels. This work presents a relatively higher pixel pitch due to the in-pixel additional circuitry. The fill factor can be improved in different ways; for example: (1) the additional amplification stages can be omitted; and (2) the digital block of the N-path filter can be moved outside the pixel by, making it shared by a higher number of pixels. A smaller capacitance $C_{R}$ can be used instead of 40 fF in order to use smaller capacitance $C_{P}$ for the same Q factor. In addition, the more advanced technologies than 130 nm have higher MOS capacitance per unit area. Thus, the layout footprint of the filter can be dramatically reduced without degrading the Q factor taking advantage of CMOS technology down-scaling.

## 8. Conclusions

This work introduces a noise reduction mechanism for CMOS THz focal plane arrays that consists of modulating the THz radiation at the source level and performing highly selective filtering at the pixel level. The noise reduction is achieved by rejecting the low frequency noise and drastically reducing the bandwidth of the thermal noise. A 31×31 pixels THz imager fabricated in a 130-nm CMOS standard process has been presented. The imager pixels include a bow tie antenna, a low noise amplifier and a high-Q filter. The latter consists of a passive SC N-path filter followed by a CT G${}_{m}$-C filter. A simplified analytical calculation of the filter transfer function is provided. The test and characterization of the presented chip validates the theoretical analysis of the filter. A high Q of 100 has been reached. The input-referred readout noise has been measured to be as low as 0.2μV RMS, and a drastic readout noise reduction has been demonstrated as expected theoretically. The noise reduction scheme has been validated by testing the imager with different THz sources in the [200–600 GHz] range. A total NEP as low as 0.6 nW at 270 GHz has been measured. The results presented with this test chip can be improved at the both sensitivity and power consumption levels. Indeed, the fill factor of the presented pixel can be improved by omitting the additional gain stages, as well as the digital blocks. The presented readout chain can be used with a more performant antenna with a better responsivity. The noise reduction of the readout chain relies on the very high-Q factor of the filter, which is directly related to the area of the MOS capacitors. Thus, the design can also be improved by using a more advanced CMOS process with a higher oxide capacitance density that would allow the same selectivity for a smaller area in order to increase the fill factor.

## Figures and Tables

**Table 1:** Overview of the presented THz imager performance compared to recently reported CMOS THz imagers.

	This Work	Reference [12]	Reference [11]
Process	130 nm CMOS	65 nm CMOS	130 nm CMOS
Array	31×31 pixels	32× 32 pixels	8×8 pixels
Pixel Pitch	240μm ×240μm	80μm ×80μm	83μm ×83μm
Power Consumption	174μW/pixel	2.5μW/pixel	150μW/pixel
Detection Device	Bow tie antenna and MOSFET rectifier	Ring antenna and MOSFET rectifier	Patch antenna and MOSFET rectifier
Responsivity $R_{v}$	300 kV/W@ 270 GHz	115 kV/W@860 GHz	3.4 kV/W@820 GHz
including on-chip gain of	58 dB	50 dB	13.5 dB
Pixel and readout chain design	Closed loop adjustable gain	Open loop gain	Open loop gain
	High selective filtering Q=100		
NEP	18.7 pW/$\sqrt{Hz}{\;}^{a}$	100 pW/$\sqrt{Hz}$	15.5 pW/$\sqrt{Hz}$
$NEP_{total}$	0.6 nW	12 nW	

${}^{a}$ The NEP measured at the center frequency of the filter.

## References

[1] Liu H.B., Zhong H., Karpowicz N., Chen Y., Zhang X.C. (2007). Terahertz Spectroscopy and Imaging for Defense and Security Applications. IEEE Proc..

[2] Yakovlev E., Zaytsev K., Dolganova I., Yurchenko S. (2015). Non-Destructive Evaluation of Polymer Composite Materials at the Manufacturing Stage Using Terahertz Pulsed Spectroscopy. IEEE Trans. Terahertz Sci. Technol..

[3] Taylor Z., Singh R., Bennett D., Tewari P., Kealey C., Bajwa N., Culjat M., Stojadinovic A., Lee H., Hubschman J. (2011). THz Medical Imaging: in vivo Hydration Sensing. IEEE Trans. Terahertz Sci. Technol..

[4] Kim J.Y., Boenawan R., Ueno Y., Ajito K. (2014). Quantitative Mapping of Pharmaceutical Cocrystals within Cellulose by Terahertz Spectroscopy. J. Lightwave Technol..

[5] Dworak V., Augustin S., Gebbers R. (2011). Application of Terahertz Radiation to Soil Measurements: Initial Results. Sensors.

[6] Han S.T., Park W.K., Chun H.S. Development of Sub-THz gyrotron for real-time food inspection. Proceedings of the 36th International Conference on Infrared, Millimeter and Terahertz Waves (IRMMW-THz).

[7] Friederich F., von Spiegel W., Bauer M., Meng F., Thomson M., Boppel S., Lisauskas A., Hils B., Krozer V., Keil A. (2011). THz Active Imaging Systems With Real-Time Capabilities. IEEE Trans. Terahertz Sci. Technol..

[8] Simoens F., Meilhan J., Nicolas J.A. (2015). Terahertz Real-Time Imaging Uncooled Arrays Based on Antenna-Coupled Bolometers or FET Developed at CEA-Leti. J. Infrared Millim. Terahertz Waves.

[9] Han R., Zhang Y., Kim Y., Kim D.Y., Shichijo H., Afshari E., Kenneth K. (2013). Active Terahertz Imaging Using Schottky Diodes in CMOS: Array and 860-GHz Pixel. IEEE J. Solid State Circuits.

[10] Schuster F., Videlier H., Dupret A., Coquillat D., Sakowicz M., Rostaing J., Tchagaspanian M., Giffard B., Knap W. A broadband THz imager in a low-cost CMOS technology. Proceedings of the 2011 IEEE International Solid-State Circuits Conference Digest of Technical Papers (ISSCC).

[11] Kim D.Y., Park S., Han R., Kenneth K. 820-GHz imaging array using diode-connected NMOS transistors in 130-nm CMOS. Proceedins of the 2013 Symposium on VLSI Circuits (VLSIC).

[12] Al Hadi R., Sherry H., Grzyb J., Zhao Y., Forster W., Keller H., Cathelin A., Kaiser A., Pfeiffer U. (2012). A 1 k-Pixel Video Camera for 0.7-1.1 Terahertz Imaging Applications in 65-nm CMOS. IEEE J. Solid State Circuits.

[13] Ghaffari A., Klumperink E., Soer M., Nauta B. (2011). Tunable High-Q N-Path Band-Pass Filters: Modeling and Verification. IEEE J. Solid State Circuits.

[14] Boukhayma A., Rostaing J.P., Mollard A., Guellec F., Benetti M., Ducournau G., Lampin J.F., Dupret A., Enz C., Tchagaspanian M. A 533pW NEP 31 × 31 pixel THz image sensor based on in-pixel demodulation. Proceedings of the European Solid State Circuits Conference (ESSCIRC), ESSCIRC 2014—40th.

[15] Dyakonov M., Shur M. (1996). Detection, mixing, and frequency multiplication of terahertz radiation by two-dimensional electronic fluid. IEEE Trans. Electron. Devices.

[16] Dyakonov M., Shur M. (1993). Shallow water analogy for a ballistic field effect transistor: New mechanism of plasma wave generation by dc current. Phys. Rev. Lett..

[17] Lisauskas A., Pfeiffer U., ÃŰjefors E., BolÃňvar P.H., Glaab D., Roskos H.G. (2009). Rational design of high-responsivity detectors of terahertz radiation based on distributed self-mixing in silicon field-effect transistors. J. Appl. Phys..

[18] Ojefors E., Pfeiffer U., Lisauskas A., Roskos H. (2009). A 0.65 THz Focal-Plane Array in a Quarter-Micron CMOS Process Technology. IEEE J. Solid State Circuits.

[19] Knap W., Kachorovskii V., Deng Y., Rumyantsev S., LÃij J.Q., Gaska R., Shur M.S., Simin G., Hu X., Khan M.A. (2002). Nonresonant detection of terahertz radiation in field effect transistors. J. Appl. Phys..

[20] Enz C., Temes G. (1996). Circuit techniques for reducing the effects of op-amp imperfections: Autozeroing, correlated double sampling, and chopper stabilization. Proc. IEEE.

[21] Schuster F., Coquillat D., Videlier H., Sakowicz M., Teppe F., Dussopt L., Giffard B., Skotnicki T., Knap W. (2011). Broadband terahertz imaging with highly sensitive silicon CMOS detectors. Opt. Express.

[22] Thomas R., Jenkins M. (1980). Analog Switches and Their Applications.

[23] Krummenacher F., Joehl N. (1988). A 4-MHz CMOS continuous-time filter with on-chip automatic tuning. IEEE J. Solid State Circuits.

[24] von Grunigen D., Sigg R., Schmid J., Moschytz G., Melchior H. (1983). An integrated CMOS switched-capacitor bandpass filter based on N-path and frequency-sampling principles. IEEE J. Solid State Circuits.

[25] Darvishi M., van der Zee R., Nauta B. (2013). Design of Active N-Path Filters. IEEE J. Solid State Circuits.

